# Genome-Wide Analysis Reveals Diverged Patterns of Codon Bias, Gene Expression, and Rates of Sequence Evolution in *Picea* Gene Families

**DOI:** 10.1093/gbe/evv044

**Published:** 2015-03-05

**Authors:** Amanda R. De La Torre, Yao-Cheng Lin, Yves Van de Peer, Pär K. Ingvarsson

**Affiliations:** ^1^Department of Ecology and Environmental Science, Umeå University, Sweden; ^2^Department of Plant Systems Biology, VIB, and Department of Plant Biotechnology and Bioinformatics, Ghent University, Ghent, Belgium; ^3^Genomics Research Institute, University of Pretoria, South Africa; ^4^Umeå Plant Science Centre, Umeå, Sweden

**Keywords:** gene expression, gene duplication, single-copy genes, codon usage, sequence divergence

## Abstract

The recent sequencing of several gymnosperm genomes has greatly facilitated studying the evolution of their genes and gene families. In this study, we examine the evidence for expression-mediated selection in the first two fully sequenced representatives of the gymnosperm plant clade (*Picea abies* and *Picea glauca*). We use genome-wide estimates of gene expression (>50,000 expressed genes) to study the relationship between gene expression, codon bias, rates of sequence divergence, protein length, and gene duplication. We found that gene expression is correlated with rates of sequence divergence and codon bias, suggesting that natural selection is acting on *Picea* protein-coding genes for translational efficiency. Gene expression, rates of sequence divergence, and codon bias are correlated with the size of gene families, with large multicopy gene families having, on average, a lower expression level and breadth, lower codon bias, and higher rates of sequence divergence than single-copy gene families. Tissue-specific patterns of gene expression were more common in large gene families with large gene expression divergence than in single-copy families. Recent family expansions combined with large gene expression variation in paralogs and increased rates of sequence evolution suggest that some *Picea* gene families are rapidly evolving to cope with biotic and abiotic stress. Our study highlights the importance of gene expression and natural selection in shaping the evolution of protein-coding genes in *Picea* species, and sets the ground for further studies investigating the evolution of individual gene families in gymnosperms.

## Introduction

Understanding the molecular changes underlying phenotypic differences between species is of great biological interest (Hahn et al. 2007). Functional evolutionary innovations leading to new phenotypes often result from changes in gene expression (Gu et al. 2004; Gallego-Romero et al. 2012; Wang et al. 2012; Jacquemin et al. 2014). Changes in gene expression are common between genes and reflect the differences in time and energy allocated to the expression of genes whose products are required by the cell in different concentrations (Williford and Demuth 2012). The more “connected” (in a network of dependency) the gene products are, the more sensitive the phenotype is to changes in product concentration (altering an individual’s fitness) (Freeling 2009; Birchler and Veitia 2012). Gene expression divergence has been linked to several gene features such as evolutionary rates, codon bias, intron size, coding sequence length, and amino acid composition. The widespread occurrence of these associations across different phylogenetic taxa highlights the role of gene expression in the evolution of protein-coding genes (Williford and Demuth 2012; Warnefors and Kaessmann 2013).

Gene expression divergence has also been linked to gene duplication. (Ohno 1970; Lynch and Katju 2004; Conant and Wolfe 2008; Flagel and Wendel 2009). Gene copies resulting from duplication may evolve under fewer functional constraints and relaxed purifying selection and eventually acquire a new function (neofunctionalization). Alternatively, the functions originally performed by an ancestral gene may be partitioned between two descendant gene copies, resulting in genes having complementary roles (subfunctionalization). The maintenance of duplicated genes may also be explained by dosage balance, network connectivity, and multiprotein complex issues (Birchler et al. 2001; Papp et al. 2003; Veitia 2004). The dosage model states that any successful genome has evolved, by positive selection, an optimum balance of gene products that interact with each other to form protein complexes, involved in multiple steps of biological processes. This model has been used to explain the maintenance of duplicates in *Arabidopsis* (Blanc and Wolfe 2004; Freeling and Thomas 2006; Barker et al. 2008; Freeling 2008) and yeast (Davis and Petrov 2005) and to justify the presence of dosage-sensitive modifiers of the white eye color in *Drosophila* (Birchler et al. 2001). In contrast, duplication of some genes may be strongly deleterious because an increase in copy number may unbalance their interactions with other proteins within the cell, leading to dosage imbalance (Makino and McLysaght 2010; De Smet et al. 2013). Because of these selective and functional constraints, single-copy genes are expected to evolve more slowly than genes in multigene families, a view widely supported by numerous studies (Han et al. 2009; Jaillon et al. 2009; De Smet et al. 2013) although a few studies have reported the opposite trend (Yang et al. 2003; Jordan et al. 2004). The relative proportions of neofunctionalization, subfunctionalization, and pseudogenization may influence gene family sizes and the evolution of gene families (Chen et al. 2010).

Several properties of gene interaction networks (such as node connectivity and centrality) may also influence gene duplicability, and may reflect differential selective forces acting on various genes (Hahn et al. 2004; Ramsay et al. 2009). Duplicated genes are found more often at the periphery of networks in *Escherichia coli*, yeast, and *Drosophila*; whereas in humans, they tend to occupy the most central positions (Doherty et al. 2012). Whole-genome studies have shown that highly connected network elements (e.g., nodes) tend to be more functionally constrained than nodes with fewer connections in both metabolic and protein–protein interaction networks (Hahn et al. 2004; Vitkup et al. 2006). Therefore, the position of an element in a network certainly affects its evolutionary fate. Upstream genes are generally more selectively constrained than downstream genes in biochemical pathways because mutations in upstream genes would generate greater pleiotropic effects potentially leading to deleterious effects (Otto 2004; Alvarez-Ponce et al. 2009; Ramsay et al. 2009). However, some studies have failed to detect a correlation between pathway position and sequence divergence (Yang et al. 2009; Jovelin and Phillips 2011).

Gymnosperms are a major plant clade that diverged from angiosperms about 300 Ma (Leslie et al. 2012). Despite initial attempts to understand their characteristic biology and unique genome architecture, studies in gymnosperms were hampered by the lack of reference genomes. The recent genome sequencing of three gymnosperms has revealed different features compared with other plant genomes (De La Torre et al. 2014). In brief, it is been shown that the enormous size of conifer genomes (20–40 Gb), by far the largest genomes sequenced to date, is not a consequence of whole-genome duplications nor it is due to an increased number of protein-coding genes. Instead, conifer genomes have grown by a slow and steady accumulation of a diverse and large set of transposable elements (Hamberger et al*.* 2009; Nystedt et al*.* 2013; De La Torre et al. 2014). Although the number of protein-coding genes is not significantly higher in gymnosperms than in angiosperms, recent phylogenetic studies have shown that some gene families have evolved differently in these two plant clades (Hamberger and Bohlmann 2006; Porth et al. 2011; Nystedt et al. 2013; Neale et al. 2014). Previous studies investigating the selective forces and evolutionary rates in gymnosperms have been based on very few numbers of genes (Willyard et al. 2007; Palme et al. 2009; Chen et al. 2010). More recently, two studies included orthologous comparisons between *Picea* and *Pinus* species using a higher number of genes (3,000–5,000) obtained from transcriptome and expressed sequence tag (EST) data (Buschiazzo et al. 2012; Chen et al. 2012). The incipient state of knowledge on the evolution of gymnosperm gene families would surely be enhanced by genome-wide studies that include analyses of gene expression data and sequence divergence in gene families of several species.

In this study, we examine the evidence for expression-mediated selection in the first two fully sequenced representatives of the gymnosperm plant clade (*Picea abies* and *Picea glauca*). We use genome-wide estimates of gene expression to investigate the relationship between gene expression, codon bias, rates of sequence divergence, and protein length. We also tested for the relationship between gene expression and gene duplication, using gene family size as a proxy; and pathway position and gene duplication (using the terpenoid pathway as an example). This study highlights the importance of gene expression and natural selection in shaping the evolution of protein-coding genes in *Picea* species.

## Materials and Methods

### Sequence Retrieval and Expression Profiles

Coding sequences were obtained for 26,597 genes from the high-confidence gene set in the *P. abies* genome (Nystedt et al. 2013; http://congenie.org, last accessed March 2015). Following the same procedure, coding sequences from 27,721 genes (derived from full-length cDNA) were retrieved from the *P. glauca* gene catalog (Rigault et al. 2011; http://www.arborea.ulaval.ca).

Expression profiles for 23,854 *P. glauca* genes were obtained for eight different tissue types, including vegetative buds, needles, xylem (mature), xylem (juvenile), phelloderm, adventitious roots, megagametophytes, and embryonic cells, from the PiceaGenExpress database (Raherison et al. 2012). Samples were collected from clonal replicates of young *P. glauca* trees in Canada. RNA was extracted, labeled, and hybridized using microarrays, as fully described in Raherison et al. (2012). Using customized Perl scripts, these genes were matched with those in the *P. glauca* gene catalog. Functional annotations were based on the detection of Pfam domains and on matches with *Arabidopsis* (TAIR 9 release) with *e* value <1e^-10^. BLAST2GO v.2.7.0 was used to perform a BLASTx search (*e* value < 1e^-10^) and Gene Ontology mapping with the plant GO-Slim terms (Conesa et al. 2005).

Expression profiles from *P. abies* were obtained from 22 samples for 8 different tissues that included needles, male and female cones, shoots, buds, pineapple galls, stems, and early and late wood. Samples were collected from multiple, pooled biological samples obtained from clonal copies growing in northern Sweden. After RNA extraction, paired-end RNA sequencing was performed using the Illumina HiSeq 2000 platform. All details of the sequencing, de novo transcriptome assembly and generation of transcripts’ expression values are fully described in Nystedt et al. (2013). Genes were ranked based on their average signal intensities within a tissue type and divided into ten equally large classes (10% quantiles) to allow comparisons with the *P. glauca* gene expression data. Expression breadth, defined as the number of tissues where a gene scored at least one hit, was also calculated for both *P. glauca* and *P. abies*. Total coding sequence length was calculated using the fastalength program from the exonerate package (version 2.2.0; Slater and Birney 2005).

### Identification of Orthologous Groups and Alignments

Open reading frames (ORF) were predicted from the cDNAs in the *P. glauca* gene catalog using the program FrameDP (Gouzy et al*.* 2009). Some redundancy was found when estimating the ORF as 2,197 cDNAs matched to more than one ORF. In these cases, the ORF with the longest sequences were kept. After ORF prediction and untranslated region (UTR) removal, 19,057 coding sequences (from an initial set of 27,721) were kept and used for posterior gene family analysis. In *P. abies*, we did not detect any ORF redundancy as we only used the “high-confidence” genes identified in the *P. abies* genome paper (Nystedt et al. 2013). A few genes (273 coding sequences), however, had partial initial codons, likely caused by partial gene lengths. This reading frame shift was corrected with an in-house BioPerl script. After ORF detection, UTR removal, and frame correction 26,164 sequences were kept from an original data set of 26,597 sequences.

We used an all-against-all BLASTP followed by a Markov Cluster algorithm to group (putative) orthologous protein sequences between the genomes of *P. glauca* and *P. abies* with the program Ortho-MCL (Li et al. 2003; http://orthomcl.org). These Ortho-MCL orthologous groups (OG) or “gene families” were composed by orthologs (between species) and recent paralogs (within species). The Ortho-MCL clustering was used to estimate gene family size. We grouped families of similar sizes according to the number of genes in each OG as follows: Single-copy (one gene in each species), 2–5 genes, 5–10 genes, 10–20 genes, 20–100 genes, and more than 100 genes. Annotation for orthologous gene families was based on Pfam domain information. Because Ortho-MCL results may be susceptible to the choice of the inflation parameter, which controls the OG size, and to the accuracy of the alignments; we manually revised the alignments of all gene families and constructed phylogenetic trees to assess whether the genes in each OG could be considered true orthologs.

In addition to Ortho-MCL, we used MUSCLE (Edgar 2004) to generate multiple alignments for each gene family. Gene families having more than 500 orthologous genes (for both *Picea* species) did not align well in MUSCLE because the similarity in the conserved domain of genes was very high whereas outside this region the similarity decreased significantly. Under these circumstances, the global multiple alignments inferred by MUSCLE were not reliable, and we therefore decided to exclude all alignments and codeml results for four very large families due to this problem. Alignments containing a majority of gaps and missing data were not considered for subsequent analyses. Non-unambiguously aligned regions in the alignment were removed based on BLOSUM62 (Henikoff S and Henikoff JG 1992) scoring matrix values allowing each aligned amino acid position with 10% of divergence, and converted to Phylip format.

### Estimation of Codon Bias

Codon bias, measured as the frequency of optimal codons (Fop), was obtained for 26,052 genes in *P. abies* and for 19,056 genes in *P. glauca* using the program CodonW (version 1.4.2, http://codonw.sourceforge.net). Only trimmed coding sequences (excluding UTRs) were analyzed. Differences in codon usage between highly expressed and lowly expressed genes in both *P. glauca* and *P. abies* were based on comparing the positions of each codon on the first and second axes of a correspondence analysis of synonymous codon usage. The identification of codons that are preferentially used in highly expressed genes (optimal codons) was done by observing the clustering of codons along the first axis (correlated with gene expression) in the correspondence analysis (supplementary figs. S1 and S2, Supplementary Material online). This set of optimal codons was then used as input in CodonW to estimate measures of codon adaptation index (CAI) and codon bias index (CBI). GC content and GC content at third position (GC3s) were also calculated with CodonW.

### Estimation of Substitution Rates

Transcript sequences for *P. glauca* and *P. abies* were trimmed and only the coding part was kept during the pairwise alignment. Based on the Ortho-MCL results, we built a list of pairwise sequences for each gene family. Then, we aligned the mRNA sequences per codon using Needle (http://www.ebi.ac.uk/Tools/psa/emboss_needle) to obtain the input files for the codeml analysis. Needle uses the Needleman–Wunsch dynamic programming algorithm to globally align two protein or nucleotide sequences along their length. Alignments containing a majority of gaps and missing data were not considered for subsequent analyses.

Synonymous (d*S*) and nonsynonymous (d*N*) nucleotide substitution rates per site were calculated using the maximum-likelihood method of Goldman and Yang (1994) in the Codeml program from the PAML package (version 4.6; Yang 2007). For each sequence pair, only the results with the highest ln *L* (log likelihood) were retained. Average scores of retained values were taken after ten repeats. The synonymous/nonsynonymous ratio (d*N*/d*S* or ω) is a measure of natural selection acting on the protein, in which values of ω < 1 mean negative purifying selection; ω = 1, neutral evolution; and ω > 1, positive selection. We discarded genes with d*S* values lower than 0.01, as these values may result in inaccurate estimates of ω, and genes with d*S* or d*N* > 2 which suggest saturation of substitutions. Abnormally high ω ratios (ω > 10) were also discarded (Villanueva-Cañas et al. 2013).

### Functional Enrichment Analyses

We tested for overrepresentation of functional categories in two of the different gene family sets (single-copy gene families and large gene families with more than 100 genes) using the BINGO 2.44 Cytoscape plugin (Maere et al. 2005). Corrections for multiple testing were done using the Benjamini and Hochberg method (1995) with a false discovery rate threshold of 0.05.

### Statistical Analyses

Expression level and expression breadth were tested for correlations with all variables including codon bias (Fop, CBI, and CAI), GC content (GC and GC3s), protein length, number of synonymous, nonsynonymous substitutions and their ratio (d*N*, d*S*, and ω), and total number of introns. Kruskal–Wallis one-way analysis of variance by ranks, which is a nonparametric test suitable for comparing groups of unequal size, was used to test associations between family size and gene expression, codon bias and rates of sequence divergence. All analyses were made using the R statistical package (version 3.0.3, R Core Team 2014).

### Correlations of Substitution Rates, Codon Bias, and Gene Expression with Pathway Position

Protein sequences involved in the conversion to lutein, abscisic acid, gibberellic acid, and brassinosteroids in the terpenoid pathway were analyzed. We used an all-against-all BLASTP followed by a Markov Cluster algorithm in Ortho-MCl, to identify orthologous protein sequences of *Arabidopsis* (as reported in Ramsay et al. 2009) in the genome of *P. abies*. Pathway position was measured following Ramsay et al.’s (2009) pathway pleiotropic index, in which groups of enzymes are numbered relative to pathway branch points from most upstream to most downstream. Correlations with codon bias (Fop), substitution rates (ω), gene expression level, and gene expression breadth were tested using the R statistical package (version 3.0.3, R Core Team 2014).

## Results

### Gene Expression

Because the number of different tissues a gene is expressed in influences gene expression, we ranked the genes based on their average signal intensities within a tissue type, to avoid possible biased correlations between codon bias and gene expression. Despite this, our analysis of 26,597 genes in *P. abies* and 27,721 genes in *P. glauca* suggests that both expression level and expression breadth were strongly correlated in *P. glauca* (*r* = 0.79, *P* < 0.001) and *P. abies* (*r* = 0.56, *P* < 0.001), as it is been reported in other species such as *Populus tremula* (Ingvarsson 2007). Expression breadth was positively correlated with protein length, and total intron length in *P. abies* (*r* = 0.17, *P* < 0.001; and *r* = 0.21, *P* < 0.001). Expression level was only weakly correlated with GC content in both *P. abies* and *P. glauca* (*r* = 0.08, *P* < 0.001; and *r* = 0.07, *P* < 0.001), but not correlated with GC content at third position in neither *P. abies* nor *P. glauca* (*r* = 0.01, *P* = 0.3705; and *r* = −0.01, *P* = 0.3596). Results of the correlations among all variables can be found in supplementary tables S1 and S2, Supplementary Material online.

### Codon Bias

Codon bias, defined as the preferential use of a subset of synonymous codons, for optimal translational efficiency, is most pronounced in highly expressed genes in species whose effective population size is large, such as many tree species (Ingvarsson 2008). In our study, we found high levels of codon bias, measured as Fop; Fop averaged 0.58 across 19,057 genes in *P. glauca*; and 0.60 across 26,164 genes in *P. abies*. Codon bias (Fop) was highly and negatively correlated with GC content and GC content at third position in both *P. glauca* (*r* = −0.78, *P* < 0.001 and *r* = −0.98, *P* < 0.001, respectively) and *P. abies* (*r* = −0.81, *P* < 0.001 and *r* = −0.98, *P* < 0.001). This suggests that optimal codons in *Picea* do not usually have G or C at the third codon position, which differs from other plant species (Serres-Giardi et al. 2012). GC content and GC at third position were also highly correlated in both species (*r* = 0.76, *P* < 0.001 in *P. glauca* and *r* = 0.79, *P* < 0.001 in *P. abies*).

**Fig. 1.—:**
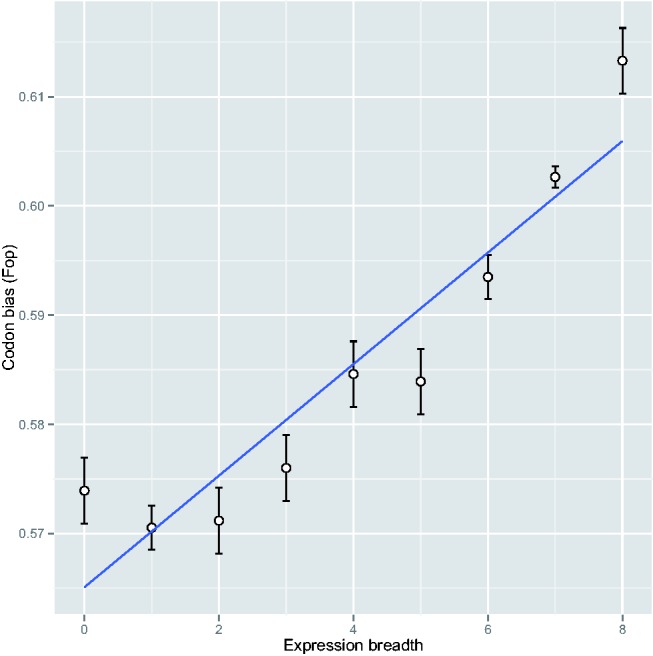
Correlation between codon bias (Fop) and expression breadth in *P. glauca*. Genes were divided into groups based on total expression breadth and average Fop was calculated for each class. Standard errors of the means were plotted using vertical lines.

There was a significant correlation between expression breadth and Fop in *P. abies* (*r* = 0.11, *P* < 0.001) and *P. glauca* (*r* = 0.14, *P* < 0.001). When genes were grouped based on their total expression breadth and average Fop was calculated for each class, the correlation between Fop and expression breadth increased significantly in *P. glauca* (*r* = 0.93, *P* < 0.001; fig. 1). In contrast, Fop was not correlated with gene expression, when gene expression was based on average signal intensities within a tissue type and divided in classes; but it was weakly correlated to gene expression when maximum gene expression across tissues was used (*r* = 0.03, *P* < 0.001).

Significant clustering of codons along the first axis of the corresponding analysis was observed in both *P. glauca* and *P. abies*. Codons in highly expressed genes were located in one extreme and codons of lowly expressed genes in the other extreme of the first axis (supplementary figs. S1 and S2, Supplementary Material online). This is usually taken as evidence that the major trend driving codon usage is correlated with gene expression, supporting the choice for optimal codons. In addition, the fact that optimal codons are the same in both spruce species (with the exception of optimal codons coding for arginine [Arg] amino acid) further supports the selection of optimal codons. The similarity in codon usage for both spruce species is consistent with the relatively shallow phylogenetic distance between species (species divergence was approximately 14 Ma). Patterns of codon usage are shown in supplementary tables S3 (*P. abies*) and S4 (*P. glauca*), Supplementary Material online.

### Rates of Sequence Divergence

Synonymous (d*S*) and nonsynonymous (d*N*) nucleotide substitution rates per site were calculated for all *P. glauca* and *P. abies* genes. The median number of synonymous substitutions (d*S*) across all gene pair combinations was 0.086 and 0.056 for nonsynonymous substitutions (d*N*). Even though the synonymous/nonsynonymous ratio (ω) varied widely among gene pair combinations, the median ratio was equal to 0.404, suggesting very strong functional constraint at most of the genes. We found 9,126 genes with a ratio (ω) lower than 1, suggesting negative purifying selection; and 1,054 genes with a ratio higher than 1, suggesting positive selection. Overrepresented functional categories of genes under diversifying selection (ω > 1) included genes involved in biotic and abiotic stress responses (leucine-rich repeat [LRR], late embryogenesis abundant, actin, histone, pollen allergen, salt stress response, stress responsive, heat repeats, auxins, dehydrins and heavy-metal associated domain, protein kinases, and cytochrome P450); and genes involved in carbohydrate metabolism and transport (several families of glycosyl hydrolases including cellulase and chitinase, and several families of glycosyl transferases) (supplementary table S7, Supplementary Material online). Similar overrepresented functional categories of genes involved in biotic and abiotic stress response were found in a smaller scale study comparing EST data between *Picea sitchensis* and *Pinus taeda* (Buschiazzo et al. 2012).

In relation to putative pseudogenes, we found 31 genes with ω > 1 that were not expressed in any tissue, and 80 genes for which gene expression data was not available in *P. glauca*. In *P. abies*, we found 30 genes with ω > 1 that lack expression data. These genes, however, had start and end codons in their coding sequences, and matched known genes in other species (http://congenie.org). Considering that we tested over 50,000 genes in this study, the number of putative pseudogenes in the data set is likely negligible, and is therefore unlikely to bias any of our results.

### Gene Family Analysis

A total of 38,662 genes (22,972 from *P. abies* and 15,690 from *P. glauca*) from both species were assigned to 5,151 OG (gene families). The frequency distribution of gene family sizes follows power-law distributions that tend to become flatter as the number of genes in the genomes increased (Huynen and van Nimwegen 1998). Most of the genes were clustered in small families, containing 2–10 genes; or belonging to single-copy gene families (supplementary fig. S3, Supplementary Material online). We define single-copy genes as those that are present in both *P. glauca* and *P. abies* and that possess a one-to-one orthologous relationship in these genomes, meaning that they have remained single-copy since their last common ancestor (approximately 14 Ma), or that have been restored to single-copy status following gene duplication during that time.

We identified a few families that contained more than 100 genes. *Picea abies* had eight families with 100–200 genes, six families with 200–300 genes, and two families with 463 and 523 genes, respectively. *Picea glauca* had five families with 100–200 genes, and one with 269 genes. Based on their Pfam annotations, these very large gene families were mainly composed of protein kinases, LRRs, and PPR (pentatricopeptide repeats). We also identified some gene families that lack orthologous gene families in the other species (740 in *P. abies* and 331 in *P. glauca*). These lineage-specific families, also called “orphans,” may arise from duplication of previously existing genes followed by rapid divergence or by de novo evolution of new genes (Heinen et al. 2009, Carvunis et al. 2012; Neme and Tautz 2014). Alternatively, they may just be artifacts of the threshold used for clustering or a result of missed annotations of genes in incompletely sequenced genomes (Hahn et al. 2007; Tautz and Domazet-Lošo 2011). The smaller number of *P. glauca*-specific gene families may be due to the incomplete nature of FL-cDNA data. In addition, 3,625 genes in *P. abies* and 3,368 genes in *P. glauca* could not be grouped using Ortho-MCL.

### Gene Family Size Is Correlated with Gene Expression, Rates of Sequence Divergence, and Codon Bias

**Fig. 2.—:**
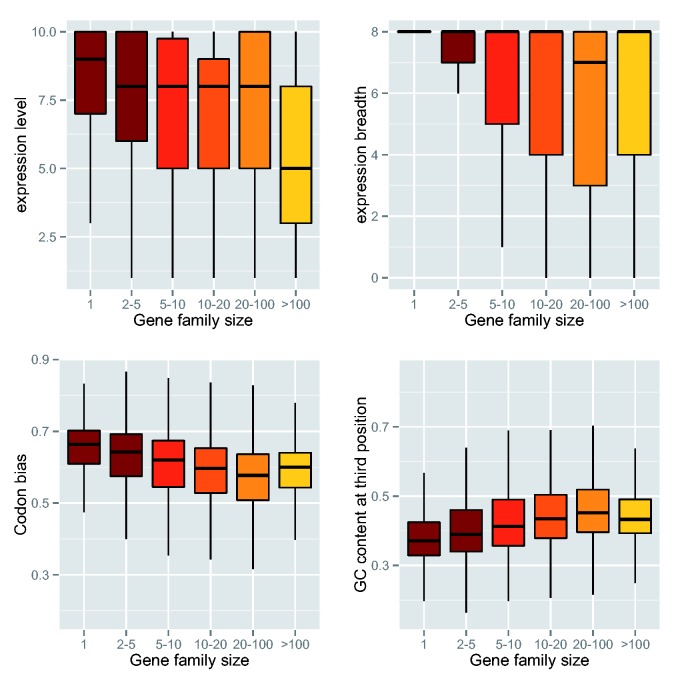
Gene family size showed significant associations with expression level, expression breadth, GC content at third position, and codon bias (Fop) in *P. abies*.

Significant correlations between family size and all variables studied, including gene expression, rates of sequence divergence (d*N*, d*S*, and ω), codon bias (Fop, CAI, CBI), GC content, and protein length were found in both *P. abies* and *P. glauca* (table 1). Expression level and expression breadth tend to decay as the size of the families increased, being the highest in single-copy gene families, and the lowest in families with over 100 genes (*P* < 2.2e^−^^16^, one-sided Mann–Whitney *U* test) (fig. 2 and supplementary fig. S4, Supplementary Material online). Interestingly, genes in the single-copy family group had the highest levels of expression breadth, meaning that they were widely expressed in most or all tissues (*P* < 2.2e^−^^16^, one-sided Mann–Whitney *U* test).

**Table 1:** Kruskal–Wallis One-Way Analysis of Variance Showed Significant Differences between Gene Families of Different Sizes for the Variables under Study

	*Picea abies*			*Picea glauca*	
Variable	df	*K*	*P* Value	*K*	*P* Value
Expression breadth	5	1,411.763	<2.2e^-16^	791.343	<2.2e^-16^
Expression level	5	1,363.149	<2.2e^-16^	175.164	<2.2e^-16^
Protein length	5	249.836	<2.2e^-16^	—	—
Fop	5	1,595.21	<2.2e^-16^	931.690	<2.2e^-16^
CBI	5	1,674.378	<2.2e^-16^	986.938	<2.2e^-16^
CAI	5	1,919.456	<2.2e^-16^	1,195.27	<2.2e^-16^
GC	5	675.907	<2.2e^-16^	411.547	<2.2e^-16^
GC3s	5	1,498.092	<2.2e^-16^	950.950	<2.2e^-16^
d*N*	5	137.538	<2.2e^-16^	489.402	<2.2e^-16^
d*S*	5	166.499	<2.2e^-16^	75.580	7.03e^-15^
ω	5	32.926	3.89e^-06^	140.641	<2.2e^-16^

Codon bias followed a similar pattern to gene expression, where families having a high expression level also had a high codon bias, due to the positive correlation between gene expression and codon bias (*P* < 2.2e^−^^16^, one-sided Mann–Whitney *U* test). With regard to protein length, single-copy gene families seemed to encode longer proteins than gene families of bigger sizes. GC content at third position increased as the size of the gene families increased (*P* < 2.2e^−^^16^, one-sided Mann–Whitney *U* test), being the lowest in single-copy gene families and the highest in families with 20–100 genes (fig. 2 and supplementary fig. S4, Supplementary Material online). Lower levels of synonymous and nonsynonymous substitutions were found in single-copy gene families than in multigene families of different sizes, with the largest families (>100 genes) having the highest substitution rates (*P* < 2.2e^−^^16^ [*P. abies*] and *P* = 4.34e^−^^7^ [*P. glauca*], one-sided Mann–Whitney *U* test) (supplementary fig. S5, Supplementary Material online).

### Functional Enrichment of Gene Families

Results of the functional enrichment analyses based on GO categories suggest very different overrepresentation of functional categories in large multicopy gene families compared with single-copy gene families (table 2). Functional enrichment in large gene families included broad molecular functions such as protein kinase activity, transferase and phosphotransferase activity, binding, catalytic activity, and signal transduction; response to different stimulus such as response to acids and bacteria; and regulation of different processes such as regulation of cellular processes, meristem growth, and immune response. It also included genes involved in reproductive development processes and postembryonic development (supplementary table S5, Supplementary Material online). Functional enrichment in single-copy gene families included genes involved in the process of gene expression such as translation; metabolism and biosynthesis of nucleotides, DNA, ncRNA, mRNA, RNA, and proteins; RNA (binding, methylation, modification, and splicing) and ncRNA processing; and nitrogen compound metabolic and biosynthetic processes (supplementary table S6, Supplementary Material online).

**Table 2:** Functional Categories Showing Overrepresentation in Large (>100 genes) and Single-Copy Gene Families in *Picea*

GO Term	GO-ID	Adjusted *P* Value (FDR < 0.05)	No. of *Picea* Genes in Big Families	Total No. of *Picea* Genes
*Large gene families*				
Protein kinase activity	4672	0.0000e^-100^	224	914
Phosphotransferase activity, alcohol group as acceptor	16773	0.0000e^-100^	224	999
Phosphorylation	16310	0.0000e^-100^	200	859
Kinase activity	16301	0.0000e^-100^	229	1,286
Phosphate metabolic process	6796	3.0000e^-100^	200	941
Phosphorus metabolic process	6793	4.0000e^-100^	200	943
Transferase activity, transferring phosphorus-containing groups	16772	2.0915e^-86^	230	1,566
Protein serine/threonine kinase activity	4674	3.1894e^-68^	122	449
Binding	5488	2.1444e^-49^	344	5,263
Nucleotide binding	166	1.4177e^-46^	190	1,776
*Single-copy gene families*				
Nucleobase, nucleoside, nucleotide, and nucleic acid metabolic process	6139	3.6236e^-59^	264	1,649
Cellular nitrogen compound metabolic process	34641	1.1583e^-57^	300	2,128
Intracellular	5622	2.3325e^-57^	570	6,668
Nitrogen compound metabolic process	6807	1.7355e^-56^	302	2,187
Nucleic acid metabolic process	90304	1.4867e^-53^	213	1,192
Cellular macromolecule metabolic process	44260	1.3065e^-51^	355	3,052
Cellular metabolic process	44237	6.7892e^-50^	493	5,409
Gene expression	10467	1.7948e^-49^	181	931
Macromolecule metabolic process	43170	1.9708e^-48^	368	3,346
Primary metabolic process	44238	1.2592e^-47^	469	5,056

### Correlations of Substitution Rates, Codon Bias, and Gene Expression with Pathway Position

Using a set of 473 orthologous protein sequences from *P. abies*, we found significant negative correlations between codon bias (Fop) and pathway position in the groups of enzymes involved in the conversion from glucose to abscisic acid (*r* = −0.61, *P* = 0), and glucose to gibberelic acid (*r* = −0.45, *P* = 0). Expression breadth was also negatively correlated with pathway position in the groups of enzymes involved in the conversion from glucose to brassinosteroid (*r* = −0.26, *P* = 0.0064), and to gibberelic acid (*r* = −0.21, *P* = 0.0053) (fig. 4). All other correlations with codon bias and expression breadth were not significant. Correlations with substitution rates (ω) were also not significant. Pathway position of all studied branches is detailed in supplementary figure S7, Supplementary Material online.

**Fig. 3.—:**
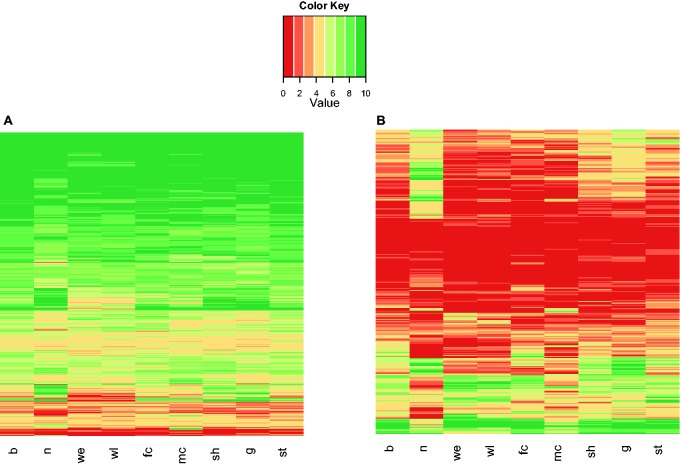
Heatmaps showing gene expression profiles in *P. abies*. (*A*) Expression profiles of orthologous single-copy genes in *P. abies*; (*B*) Heatmap showing diversified expression profiles among paralogous genes of the LRR gene family. Each row is a gene and each column is a tissue. Expression levels vary from 0 (lowly expressed) to 10 (highly expressed). Tissues studied included buds (b), needles (n), wood-early (we), wood-late (wl), female cone (fm), male cone (mc), shoots (s), galls (g), and stems (st).

## Discussion

### Gene Expression and Rates of Sequence Divergence

Although changes in gene expression may play an important role in phenotypic divergence and adaptation, the role of natural selection on the evolution of gene expression levels remains largely unknown in nonmodel species (Gilad et al. 2006; Bedford and Hartl 2009). Our study in *Picea* shows that selection is likely acting on gene expression to increase the efficiency and accuracy of transcription and protein synthesis and processing (fig. 1). Our results are further sustained by the fact that highly expressed genes may be under greater selective constraints than lowly expressed genes, based on the negative correlation between d*N*, ω, expression level and expression breadth in both *P. glauca* and *P. abies* (supplementary tables S1 and S2, Supplementary Material online). This relationship between d*N* and gene expression has also been observed in other plant species such as *Populus* and *Arabidopsis* (Ganko et al. 2007; Ingvarsson 2007). However protein length and intron sizes increased with gene expression, which is contrary to the expectation for selection to reduce transcriptional cost, and suggests that other factors may be influencing protein length in *Picea*.

The relationship between d*N* and gene expression divergence may also be interpreted as a correlation between protein divergence and expression divergence, suggesting an important role of gene expression in the evolution of protein-coding genes in *Picea* species. Our results showing that highly expressed genes are associated with slow-evolving protein sequences and less divergent gene expression patterns may suggest that the correlation between protein divergence and expression divergence is a result of between-gene variation in expression levels. However, our study also suggests that other gene characteristics such as tissue specificity, mutation rate (d*S*), gene family size, and connectivity may also contribute to explain the correlation. This suggests that the correlation between expression divergence and protein divergence is not linked to a specific gene characteristic, but instead reflects more general selective constraints, supporting recent studies in mammals (Warnefors and Kaessmann 2013).

### Gene Expression and Codon Bias

Changes in gene sequence and structure that lead to a reduction in time or energy spent in the complex processes of transcription and translation may be particularly favored in highly expressed genes (Akashi 1994; Stoletzki and Eyre-Walker 2007; Zhou et al. 2009; Williford and Demuth 2012). Codon bias, defined as the preferential use of a subset of synonymous codons, is determined by a balance between drift, mutation, and natural selection for optimal translational efficiency and/or accuracy (Akashi 2001). In species whose effective population size is large, such as prokaryotes, unicellular eukaryotes and some tree species (e.g., *Populus*), natural selection may be the main force shaping codon usage (Ingvarsson 2008, 2009). In our study in *Picea*, we found that a subset of synonymous codons is preferentially used in highly and widely expressed genes (supplementary tables S3 and S4, and figs. S1 and S2, Supplementary Material online), supporting the role of selection for translational efficiency. This significant positive correlation between gene expression and codon bias has been previously observed in several organisms including *E. coli*, *Saccharomyces cerevisiae*, *Caenorhabditis elegans*, *Arabidopsis thaliana*, *Drosophila melanogaster* (reviewed in Plotkin and Kudla 2011) and more recently in *Populus tremula* (Ingvarsson 2007), *Silene latifolia* (Qiu et al. 2011), *Cardamine* spp (Ometto et al. 2012), and *Tribolium castaneum* (Williford and Demuth 2012). Codon bias may also have a role in protein export. High-frequency of nonoptimal codons has been found in the signal sequences of the N-terminal regions of proteins exported through secretory pathways, which seems to be important for the correct folding of pre-exported proteins (Humphreys et al. 2000; Power et al. 2004; Palazzo et al. 2007; Zalucki et al. 2009).

Translational efficiency may also be influenced by modifications in the noncoding portion of the genes. Changes in the promoter region and alternative promoter usage during the process of transcription and post-transcriptional regulation may lead to transcripts exhibiting reduced or enhanced translational efficiency in plants and animals (Larsen et al. 2002; Hong et al. 2012; Huang et al. 2013). Although studying promoter regions was out of the scope of this work, our analysis of nine *Picea* families of different sizes showed a greater variation of motifs (located 1 kb upstream UTR sequences) in large gene families (showing a lower codon bias) than in single-copy and small gene families (showing a higher codon bias). More studies are needed to understand how the variation in promoter regions affects translational efficiency in gymnosperm species. An example of the most significant motifs for three of the studied gene families can be found in supplementary figures S8 and S9, Supplementary Material online.

### Gene Expression and Gene Duplication

By using gene family size as a proxy for gene duplication, we found a strong correlation between gene expression and gene duplication in the two *Picea* species, with large gene families having, on average, a lower expression level and breadth, lower codon bias, and higher rates of sequence divergence than single-copy gene families (table 1, fig. 2 and supplementary figs. S4 and S5, Supplementary Material online). A correlation between gene duplication and gene expression variation has been previously observed in a small-scale study of gene expression networks in *P. glauca,* and also in *Arabidopsis* and rice (Hanada et al. 2008; Verta et al. 2013). Our results suggest that the evolution of gene family size in *Picea* is under strong functional and selective constraints.

Gene duplication may also be influenced by the position of genes in the pathway. As an example, our study of the terpenoid metabolic pathway in *Picea* suggests that duplicated genes in large gene families are more often found in the downstream branches of the pathway, supporting previous studies in *E. coli*, yeast and *Drosophila*, but differing from those in humans (Doherty et al. 2012). These results may suggest more selective constraints in upstream genes than in downstream genes; however, we did not find a significant correlation between rates of sequence divergence (ω) and pathway position for any of the branches of the pathway studied.

**Fig. 4.—:**
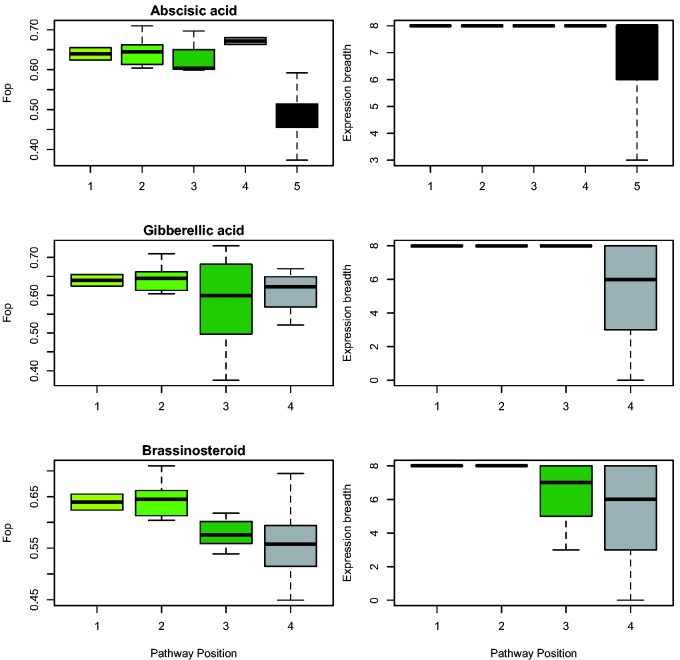
Relationship between codon bias (Fop) and expression breadth with pathway position for *P. abies* in three of the four branches of the terpenoid synthesis. Plant terpenoid simplified pathway showing the pathway positions of each of the branches can be found in supplementary figure S7, Supplementary Material online.

In contrast, we found a strong negative correlation between codon bias, and gene expression breadth with pathway position in several of the branches (fig. 4), with upstream genes having a higher codon bias and being more widely expressed than genes in downstream positions. These findings further support our previous results showing a correlation between gene expression and gene duplication, and suggest that pathway position is influencing the patterns of gene duplication in *Picea* species.

### Gene Expression in Single-Copy Gene Families

Single-copy genes may be under strong selective constraints, because an increase in copy number may unbalance their interactions with other proteins resulting in deleterious effects (Makino and McLysaght 2010; De Smet et al. 2013). Our study in *Picea* showed that there is selective pressure to maintain genes encoded ancient conserved biological functions such as translation, DNA/RNA metabolism, and nuclease activity as singletons; supporting recent studies in angiosperm species (Armisen et al. 2008; Duarte et al. 2010; Zhang et al. 2012; De Smet et al. 2013).

In addition, our results suggest that single-copy genes are widely expressed in all or most tissues and have higher expression levels, on average, than genes in multigene families (figs. 2 and 3 and supplementary fig. S4, Supplementary Material online). Gene expression of single-copy genes showed more divergence among tissues in *P. glauca* than in *P. abies*, with paralogs that are expressed in megagametophytes showing the highest expression and least diverged patterns in *P. glauca* (supplementary fig. S6, Supplementary Material online). Single-copy genes also had the lowest levels of tissue-specific gene expression in both *P. glauca* and *P. abies*, when compared with duplicated gene families (fig. 3). The comparison between single-copy orthologs, however, was limited because of the different experimental procedures used to generate the expression data and the different tissues analyzed in each of the studied species. Despite the differences, overall expression levels of *P. abies* and *P. glauca* single-copy orthologs were significantly correlated (*r* = 0.54, *P* < 0.0001).

We found that single-copy genes were longer, on average, than genes in multigene families, which agrees with the view that housekeeping and highly expressed genes are less compact than tissue-specific genes in humans and plants (Ren 2006; Zhu et al. 2008). Based on the low number of synonymous and nonsynonymous substitutions, we believe that Picea housekeeping genes may have evolved more slowly and are therefore more conserved than genes in multigene families. This is further corroborated by a recent study based on nucleotide polymorphisms that showed that genes with high expression level and breadth were more conserved than tissue-specific genes in *P. glauca* (Pavy et al. 2013). Other examples of genes under highly functional constraint on gene duplication are the genes encoding the LEAFY transcription factor, one of the few transcription factors found as single-copy in all plant species, with the exception of gymnosperms (Baum et al. 2005). The LEAFY gene constitutes a special case of single-copy gene that has evolved new DNA binding specificities through a promiscuous intermediate, without losing its initial function (Kovach and Lamb 2014; Sayou et al. 2014).

### Gene Expression Divergence in Large Gene Families

We found that large gene families in *Picea* had higher gene expression variation and higher rates of sequence divergence than genes in smaller gene families including single-copy genes. Because different copies of duplicate genes may become specialized at different times, duplicate genes may have more diversified expression profiles than single-copy genes, so that the expression patterns of duplicate genes are expected to diverge between species faster than those of single-copy genes (Gu et al. 2004). Gene expression also varied among tissues, with *Picea* paralogs having diversifying expression from tissue-specific to broadly expressed in most or all tissues. In a recent study in *Arabidopsis*, 97% of paralogous pairs showed evidence of functional diversification as a result of both neo- and subfunctionalization (Guo et al. 2013). Similarly, expression divergence of ancient paralogs leading to tissue specialization was common in *Gossypium* (Renny-Byfield et al. 2014). Successive rounds of sub- or neofunctionalization may lead to high expression variation among paralogous copies that in turn result in fitness advantages (Schmid et al. 2005). An example of this are the genes in the terpene synthase family in *Picea* which have undergone repeated rounds of neofunctionalization resulting in a broad diversity of secondary metabolites that are crucial in warding off pathogens and herbivores (Keeling et al. 2008).

Our study showed that LRR and protein kinases, which are two of the largest gene families found in conifers, have gone through recent gene family expansions in conifers and also have some of the most diversified gene expression patterns among *Picea* paralogous genes (fig. 3). Interestingly, these families also showed overrepresentation among genes with ω > 1, suggesting that they are evolving as a response to natural selection in *Picea*. Studies in *Arabidopsis* and rice suggest that the kinase family has largely evolved as a response to biotic stress and has greatly expanded as a consequence of adaptation to fast-evolving pathogens (Hanada et al. 2008; Jacquemin et al. 2014). Similarly, LRR families, which have a role in disease resistance and protein–protein interactions mediated by specific amino acids, seem to be under diversifying selection in *Arabidopsis* and *Solanum* (Flagel and Wendel 2009; Slotte et al. 2011). Recent studies have suggested that different types of LRR (e.g., toll-interleukin receptor/nucleotide binding/LRR gene) have expanded in conifers and angiosperms (Neale et al. 2014).

By using recently available genomic resources of an understudied yet important major plant clade, our study sheds light on the role of gene expression and natural selection on the evolution of protein-coding genes in gymnosperms. This work advances our current understanding of plant science by showing the complex relationships between gene expression, codon bias, rates of sequence divergence, and gene duplication in gymnosperms. Our ongoing research includes phylogenomic and molecular evolution analyses of duplicated and single-copy genes in gymnosperm species.

## Supplementary Material

Supplementary tables S1–S7 and figures S1–S9 are available at *Genome Biology and Evolution* online (http://www.gbe.oxfordjournals.org/).

Supplementary Data
