# Supplementary material for: Genome-Wide Analysis Reveals Diverged Patterns of Codon Bias, Gene Expression, and Rates of Sequence Evolution in Picea Gene Families
Source: Genome Biol Evol. 2015 Mar 5;7(4):1002–15. doi: 10.1093/gbe/evv044 (PMC4419791; doi:10.1093/gbe/evv044)
Supplement: Supplementary Data [file supp_7_4_1002__index.html]

Genome-Wide Analysis Reveals Diverged Patterns of Codon Bias, Gene Expression, and Rates of Sequence Evolution in Picea Gene Families — Supplementary Data 

# Genome-Wide Analysis Reveals Diverged Patterns of Codon Bias, Gene Expression, and Rates of Sequence Evolution in *Picea* Gene Families

## Supplementary Data

files

**Files in this Data Supplement:**

- Supplementary Data - zip file
