# Supplementary material for: Genome-Wide Analysis Reveals Diverged Patterns of Codon Bias, Gene Expression, and Rates of Sequence Evolution in Picea Gene Families
Source: Genome Biol Evol. 2015 Mar 5;7(4):1002–15. doi: 10.1093/gbe/evv044 (PMC4419791; doi:10.1093/gbe/evv044)
Supplement: Supplementary Data [file supp_evv044_suppl_data.zip › New Microsoft Office Word Document.docx]

**Supplementary Material**

Table S1. Relationships among studied variables based on 27,597 genes in *Picea abies.* Significant correlations are in bold numbers.

Table S2. Relationships among studied variables based on 25,384 genes in *Picea glauca.* Significant correlations are in bold numbers.

Table S3. Codon usage in *Picea abies.*

Table S4. Codon usage in *Picea glauca.*

Table S5. Over-representation of functional categories among *Picea* genes in large size gene families (>100 genes).

Table S6. Over-representation of functional categories among *Picea* genes in single-copy gene families.

Table S7. Genes with rates of sequence divergence higher than 1 (w>1), potentially under diversifying selection (on-line version).

Figure S1. Plot of axis 1 to 4 of a correspondence analysis of codon usage in 26,052 genes from *P. abies*. Black circles represent codons from highly expressed genes, and red circles, codons from lowly expressed genes.

Figure S2. Plot of axis 1 to 4 of a correspondence analysis of codon usage in 19,056 genes from *P. glauca*. Black circles represent codons from highly expressed genes, and red circles, codons from lowly expressed genes.

Figure S3. Frequency distribution of gene family size in *P.glauca* and *P.abies*. Each dot represents one orthologous gene family.

Figure S4. Gene family size showed significant associations with expression level, expression breadth, GC content at third position and codon bias (Fop) in *P. glauca*.

Figure S5. Number of synonymous nucleotide substitutions (dS) and non-synonymous nucleotide substitutions (dN) vs. gene family size.

Figure S6. Heatmap showing gene expression profiles of orthologous single copy genes in *P. glauca*. Each row is a single copy gene, and each column is a tissue. Expression levels go from 0 (lowly expressed) to 10 (highly expressed). Tissues studied included buds (b), needles (n), wood-early (we), wood-late (wl), megagametophytes (meg), phelloderm (phe), adventitious roots (ro) and embryonic cells (ec).

Figure S7. Plant terpenoid simplified pathway, modified from Ramsay et al. 2009. Numbers indicate pathway positions in each of the studied branches.

Figure S8. Sequence logo showing motif enrichment in the three most statistically significant motifs (from top to bottom) in the Promoter region of genes pertaining to two *Picea abies* gene families: A) Gene family “Picea 1012”, containing 118 genes, and annotated as MYb-like DNA-binding domain (PF00249.26, PF1392.1); and B) Gene family “Picea 1028”, containing 62 genes, annotated as Pectinesterase (PF01095.14; PF04043.10). The height of the motif block is proportional to –log (p-value), truncated as the height for a motif with a p-value of 1e-10. Sequence logos were obtained using MEME (Bailey et al. 2009).

Figure S9. Figure shows A) sequence logo showing motif enrichment in the three most statistically significant motifs (from top to bottom) in the Promoter region of genes pertaining to *Picea abies* gene family “Picea 1236”, containing 23 genes, annotated as Fasciclin (PF02469.17). Sequence logos were obtained using MEME (Bailey et al. 2009). B) Maximum-Likelihood phylogenetic tree with 100 bootstrap replicates showing all orthologous and paralogous genes for *P. abies* and *P. glauca*, for the same gene family “Picea 1236”. Phylogenetic tree was constructed using MEGA 6.06 (Tamura et al. 2013).
