# Supplementary material for: Genome-Wide Analysis Reveals Diverged Patterns of Codon Bias, Gene Expression, and Rates of Sequence Evolution in Picea Gene Families
Source: Genome Biol Evol. 2015 Mar 5;7(4):1002–15. doi: 10.1093/gbe/evv044 (PMC4419791; doi:10.1093/gbe/evv044)
Supplement: Supplementary Data [file supp_evv044_suppl_data.zip › Supplementary Info_Genome-wide expression.docx]

**Supplementary Information**

Table S1. Relationships among studied variables based on 27,597 genes in *Picea abies.* Significant correlations are in bold numbers.

| variable | Pearson corr | Fop | expression breadth | expression level | protein lenght | rmd | intron total | GC | GC3s | CAI | CBI | dNdS | dN | dS |
| --- | --- | --- | --- | --- | --- | --- | --- | --- | --- | --- | --- | --- | --- | --- |
| Fop | r | **1** | **0.11** | -0.01 | **0.32** | **-0.35** | **0.24** | **-0.81** | **-0.98** | **0.93** | **1** | **-0.03** | -0.01 | **0.07** |
|  | P-value | **0** | **0** | 0.0316 | **0** | **0** | **0** | **0** | **0** | **0** | **0** | **0.0087** | 0.5194 | **0** |
| expression breadth | r | **0.11** | **1** | **0.56** | **0.17** | **-0.66** | **0.21** | -0.01 | **-0.12** | **0.14** | **0.12** | **-0.03** | **-0.06** | **0.02** |
|  | P-value | **0** | **0** | **0** | **0** | **0** | **0** | 0.0458 | **0** | **0** | **0** | **0.0012** | **0** | 0.0575 |
| expression level | r | -0.01 | **0.56** | **1** | **0.03** | **-0.37** | **0.05** | **0.08** | 0.01 | **0.02** | -0.01 | -0.01 | **-0.05** | -0.03 |
|  | P-value | 0.0316 | **0** | **0** | **0** | **0** | **0** | **0** | 0.3705 | **0.0083** | 0.299 | 0.375 | **0** | 0.0146 |
| protein length | r | **0.32** | **0.17** | **0.03** | **1** | **-0.27** | **0.18** | **-0.29** | **-0.31** | **0.31** | **0.32** | -0.02 | **0.04** | **0.09** |
|  | P-value | **0** | **0** | **0** | **0** | **0** | **0** | **0** | **0** | **0** | **0** | 0.0562 | **0.0005** | **0** |
| rmd | r | **-0.35** | **-0.66** | **-0.37** | **-0.27** | **1** | **-0.32** | **0.26** | **0.34** | **-0.39** | **-0.35** | **0.04** | 0.03 | **-0.06** |
|  | P-value | **0** | **0** | **0** | **0** | **0** | **0** | **0** | **0** | **0** | **0** | **0** | 0.0151 | **0** |
| intron total | r | **0.24** | **0.21** | **0.05** | **0.18** | **-0.32** | **1** | **-0.17** | **-0.24** | **0.24** | **0.24** | **-0.04** | -0.02 | **0.03** |
|  | P-value | **0** | **0** | **0** | **0** | **0** | **0** | **0** | **0** | **0** | **0** | **0.0009** | 0.1216 | **0.007** |
| GC | r | **-0.81** | -0.01 | **0.08** | **-0.29** | **0.26** | **-0.17** | **1** | **0.79** | **-0.82** | **-0.79** | 0.01 | 0 | **-0.04** |
|  | P-value | **0** | 0.0458 | **0** | **0** | **0** | **0** | **0** | **0** | **0** | **0** | 0.4148 | 0.7064 | **0** |
| GC3s | r | **-0.98** | **-0.12** | 0.01 | **-0.31** | **0.34** | **-0.24** | **0.79** | **1** | **-0.91** | **-0.98** | **0.03** | 0.01 | **-0.07** |
|  | P-value | **0** | **0** | 0.3705 | **0** | **0** | **0** | **0** | **0** | **0** | **0** | **0.0032** | 0.4889 | **0** |
| CAI | r | **0.93** | **0.14** | **0.02** | **0.31** | **-0.39** | **0.24** | **-0.82** | **-0.91** | **1** | **0.93** | **-0.03** | -0.01 | **0.07** |
|  | P-value | **0** | **0** | **0.0083** | **0** | **0** | **0** | **0** | **0** | **0** | **0** | **0.0057** | 0.2428 | **0** |
| CBI | r | **0** | **0.12** | -0.01 | **0.32** | **-0.35** | **0.24** | **-0.79** | **-0.98** | **0.93** | **1** | **-0.03** | -0.01 | **0.07** |
|  | P-value | **1** | **0** | 0.299 | **0** | **0** | **0** | **0** | **0** | **0** | **0** | **0.0047** | 0.4751 | **0** |
| dNdS | r | **-0.03** | **-0.03** | -0.01 | -0.02 | **0.04** | **-0.04** | 0.01 | **0.03** | **-0.03** | **-0.03** | **1** | **0.20** | **-0.08** |
|  | P-value | **0.0087** | **0.0012** | 0.375 | 0.0562 | **0** | **0.0009** | 0.4148 | **0.0032** | **0.0057** | **0.0047** | **0** | **0** | **0** |
| dN | r | -0.01 | **-0.06** | **-0.05** | **0.04** | 0.03 | -0.02 | 0 | 0.01 | -0.01 | -0.01 | **0.2** | **1** | **0.05** |
|  | P-value | 0.5194 | **0** | **0** | **0.0005** | 0.0151 | 0.1216 | 0.7064 | 0.4889 | 0.2428 | 0.4751 | **0** | **0** | **0** |
| dS | r | **0.07** | **0.02** | -0.03 | **0.09** | **-0.06** | **0.03** | **-0.04** | **-0.07** | **0.07** | **0.07** | **-0.08** | **0.05** | **1** |
|  | P-value | **0** | 0.0575 | 0.0146 | **0** | **0** | **0.007** | **0** | **0** | **0** | **0** | **0** | **0** | **0** |

Table S2. Relationships among studied variables based on 25,384 genes in *Picea glauca.* Significant correlations are in bold numbers.

| variable | Pearson correlation | Fop | expression level | expression breadth | GC | GC3s | CAI | CBI | dNdS | dN | dS |
| --- | --- | --- | --- | --- | --- | --- | --- | --- | --- | --- | --- |
| Fop | r | **1** | 0 | **0.14** | **-0.78** | **-0.98** | **0.93** | **1** | -0.01 | 0 | **0.03** |
|  | P-value | **0** | 0.9705 | **0** | **0** | **0** | **0** | **0** | 0.2772 | 0.8483 | **0.001** |
| expression level | r | 0 | **1** | **0.79** | **0.07** | -0.01 | 0.02 | 0.01 | **-0.03** | **-0.03** | -0.02 |
|  | P-value | 0.9705 | **0** | **0** | **0** | 0.3596 | 0.0208 | 0.4947 | **0.0014** | **0.0085** | 0.0252 |
| expression breadth | r | **0.14** | **0.79** | **1** | **-0.06** | **-0.14** | **0.18** | **0.15** | **-0.04** | **-0.03** | 0.01 |
|  | P-value | **0** | **0** | **0** | **0** | **0** | **0** | **0** | **0** | **0.0029** | 0.2938 |
| GC | r | **-0.78** | **0.07** | **-0.06** | **1** | **0.76** | **-0.81** | **-0.77** | 0 | 0 | -0.02 |
|  | P-value | **0** | **0** | **0** | **0** | **0** | **0** | **0** | 0.8918 | 0.7155 | 0.0335 |
| GC3s | r | **-0.98** | -0.01 | **-0.14** | **0.76** | **1** | **-0.91** | **-0.98** | 0.01 | 0 | **-0.03** |
|  | P-value | **0** | 0.3596 | **0** | **0** | **0** | **0** | **0** | 0.2875 | 0.8537 | **0.0004** |
| CAI | r | **0.93** | 0.02 | **0.18** | **-0.81** | **-0.91** | **1** | **0.93** | -0.01 | 0 | **0.04** |
|  | P-value | **0** | 0.0208 | **0** | **0** | **0** | **0** | **0** | 0.2837 | 0.8 | **0** |
| CBI | r | **1** | 0.01 | **0.15** | **-0.77** | **-0.98** | **0.93** | **1** | -0.01 | 0 | **0.03** |
|  | P-value | **0** | 0.4947 | **0** | **0** | **0** | **0** | **0** | 0.1915 | 0.9281 | **0.0005** |
| dNdS | r | -0.01 | **-0.03** | **-0.04** | 0 | 0.01 | -0.01 | -0.01 | **1** | **0.19** | **-0.09** |
|  | P-value | 0.2772 | **0.0014** | **0** | 0.8918 | 0.2875 | 0.2837 | 0.1915 | **0** | **0** | **0** |
| dN | r | 0 | **-0.03** | **-0.03** | 0 | 0 | 0 | 0 | **0.19** | **1** | **0.06** |
|  | P-value | 0.8483 | **0.0085** | **0.0029** | 0.7155 | 0.8537 | 0.8 | 0.9281 | **0** | **0** | **0** |
| dS | r | **0.03** | -0.02 | 0.01 | -0.02 | **-0.03** | **0.04** | **0.03** | **-0.09** | **0.06** | **1** |
|  | P-value | **0.001** | 0.0252 | 0.2938 | 0.0335 | **0.0004** | **0** | **0.0005** | **0** | **0** | **0** |

Table S3. Codon usage in *Picea abies*

| Amino acid | Codon | Codon frequency in highly-expressed genes (Number) | Codon frequency in lowly expressed genes (Number) |
| --- | --- | --- | --- |
| Phe | UUU* | 1.49 (8898) | 0.61 (4270) |
|  | UUC | 0.51 (3056) | 1.39 (9721) |
| Leu | UUA* | 1.09 (6478) | 0.29 (1519) |
|  | UUG* | 1.56 (9298) | 1.05 (5457) |
|  |  |  |  |
|  | CUU* | 1.59 (9447) | 0.72 (3730) |
|  | CUC | 0.38 (2273) | 1.78 (9247) |
|  | CUA* | 0.70 (4161) | 0.31 (1635) |
|  | CUG | 0.68 (4034) | 1.85 (9604) |
|  |  |  |  |
| Ile | AUU* | 1.59 (9901) | 0.91 (4533) |
|  | AUC | 0.47 (2923) | 1.46 (7282) |
|  | AUA* | 0.94 (5883) | 0.63 (3145) |
| Met | AUG | 1.00 (9572) | 1.00 (7973) |
|  |  |  |  |
| Val | GUU* | 1.69 (10465) | 0.93 (5202) |
|  | GUC | 0.46 (2851) | 1.15 (6418) |
|  | GUA* | 0.93 (5785) | 0.37 (2082) |
|  | GUG | 0.92 (5686) | 1.54 (8596) |
|  |  |  |  |
| Tyr | UAU* | 1.51 (6199) | 0.74 (3197) |
|  | UAC | 0.49 (2000) | 1.26 (5393) |
| TER | UAA | 0.86 (342) | 0.92 (322) |
|  | UAG | 0.85 (339) | 0.77 (271) |
|  | UGA | 1.28 (509) | 1.31 (457) |
|  |  |  |  |
| His | CAU* | 1.54 (7630) | 0.81 (2972) |
|  | CAC | 0.46 (2286) | 1.19 (4348) |
| Gln | CAA* | 1.15 (11298) | 0.62 (3200) |
|  | CAG | 0.85 (8342) | 1.38 (7203) |
|  |  |  |  |
| Asn | AAU* | 1.50 (13731) | 0.84 (5343) |
|  | AAC | 0.50 (4576) | 1.16 (7385) |
| Lys | AAA* | 1.09 (13657) | 0.67 (4931) |
|  | AAG | 0.91 (11496) | 1.33 (9833) |
|  |  |  |  |
| Asp | GAU* | 1.56 (17971) | 0.86 (6457) |
|  | GAC | 0.44 (5080) | 1.14 (8593) |
| Glu | GAA* | 1.24 (20529) | 0.75 (6488) |
|  | GAG | 0.76 (12602) | 1.25 (10714) |
|  |  |  |  |
| Ser | UCU* | 1.77 (11677) | 0.93 (4180) |
|  | UCC | 0.52 (3439) | 1.41 (6333) |
|  | UCA* | 1.72 (11347) | 0.50 (2247) |
|  | UCG | 0.19 (1245) | 1.21 (5460) |
|  | AGU* | 1.25 (8295) | 0.50 (2260) |
|  | AGC | 0.55 (3656) | 1.45 (6527) |
|  |  |  |  |
| Pro | CCU* | 1.67 (8034) | 0.80 (3636) |
|  | CCC | 0.38 (1844) | 1.36 (6199) |
|  | CCA* | 1.80 (8682) | 0.59 (2664) |
|  | CCG | 0.15 (732) | 1.25 (5679) |
|  |  |  |  |
| Thr | ACU* | 1.50 (7587) | 0.76 (3079) |
|  | ACC | 0.44 (2240) | 1.27 (5131) |
|  | ACA* | 1.88 (9515) | 0.67 (2700) |
|  | ACG | 0.17 (884) | 1.30 (5269) |
|  |  |  |  |
| Ala | GCU* | 1.58 (11061) | 0.77 (5158) |
|  | GCC | 0.44 (3062) | 1.50 (10070) |
|  | GCA* | 1.86 (12982) | 0.64 (4314) |
|  | GCG | 0.12 (811) | 1.09 (7343) |
|  |  |  |  |
| Cys | UGU* | 1.33 (4035) | 0.56 (1787) |
|  | UGC | 0.67 (2037) | 1.44 (4631) |
|  |  |  |  |
| Trp | UGG | 1.00 (3614) | 1.00 (4811) |
|  |  |  |  |
| Arg | CGU* | 0.78 (2542) | 0.54 (1739) |
|  | CGC | 0.31 (1006) | 1.07 (3418) |
|  | CGA | 0.66 (2138) | 0.68 (2187) |
|  | CGG | 0.42 (1355) | 1.13 (3613) |
|  | AGA* | 2.24 (7270) | 1.21 (3881) |
|  | AGG* | 1.59 (5166) | 1.37 (4396) |
|  |  |  |  |
| Gly | GGU* | 1.35 (8412) | 0.59 (3522) |
|  | GGC | 0.52 (3259) | 1.44 (8667) |
|  | GGA* | 1.43 (8906) | 0.91 (5467) |
|  | GGG | 0.69 (4271) | 1.06 (6366) |

Notes:

* Codons showing significantly higher frequencies in highly- expressed genes (P<0.001).

Table S4. Codon usage in *Picea glauca*

| Amino acid | Codon | Codon frequency in highly-expressed genes (Number) | Codon frequency in lowly expressed genes (Number) |
| --- | --- | --- | --- |
| Phe | UUU* | 1.49(5547) | 0.47(1669) |
|  | UUC | 0.51(1892) | 1.53(5495) |
| Leu | UUA* | 1.18(4090) | 0.17(424) |
|  | UUG* | 1.50(5207) | 0.90(2191) |
|  |  |  |  |
|  | CUU* | 1.62(5594) | 0.75(1836) |
|  | CUC | 0.38(1317) | 2.33(5674) |
|  | CUA* | 0.71(2445) | 0.24(584) |
|  | CUG | 0.61(2126) | 1.61(3926) |
|  |  |  |  |
| Ile | AUU* | 1.63(6088) | 0.76(2104) |
|  | AUC | 0.45(1678) | 1.92(5306) |
|  | AUA* | 0.92(3447) | 0.31(869) |
| Met | AUG | 1.00(4828) | 1.00(3610) |
|  |  |  |  |
| Val | GUU* | 1.71(5603) | 0.82(2194) |
|  | GUC | 0.43(1402) | 1.75(4710) |
|  | GUA* | 0.95(3111) | 0.27(713) |
|  | GUG | 0.91(2976) | 1.17(3141) |
|  |  |  |  |
| Tyr | UAU* | 1.50(4065) | 0.47(1047) |
|  | UAC | 0.50(1337) | 1.53(3440) |
| TER | UAA | 1.06(290) | 1.03(291) |
|  | UAG | 0.75(207) | 0.91(259) |
|  |  |  |  |
| His | CAU* | 1.56(3732) | 0.58(1067) |
|  | CAC | 0.44(1057) | 1.42(2596) |
| Gln | CAA* | 1.20(4970) | 0.62(1723) |
|  | CAG | 0.80(3321) | 1.38(3879) |
|  |  |  |  |
| Asn | AAU* | 1.52(6122) | 0.53(1849) |
|  | AAC | 0.48(1951) | 1.47(5161) |
| Lys | AAA* | 1.11(6902) | 0.44(1958) |
|  | AAG | 0.89(5480) | 1.56(6976) |
|  |  |  |  |
| Asp | GAU* | 1.54(8447) | 0.66(2577) |
|  | GAC | 0.46(2509) | 1.34(5286) |
| Glu | GAA* | 1.23(9097) | 0.53(2074) |
|  | GAG | 0.77(5679) | 1.47(5736) |
| Ser | UCU* | 1.68(4482) | 0.84(1859) |
|  | UCC | 0.47(1253) | 1.81(3998) |
|  | UCA* | 1.79(4763) | 0.42(934) |
|  | UCG | 0.19(514) | 1.06(2342) |
|  | AGU* | 1.29(3439) | 0.42(924) |
|  | AGC | 0.57(1523) | 1.46(3232) |
|  |  |  |  |
| Pro | CCU* | 1.65(3526) | 0.76(1669) |
|  | CCC | 0.36(771) | 1.67(3686) |
|  | CCA* | 1.83(3909) | 0.58(1271) |
|  | CCG | 0.16(342) | 1.00(2205) |
|  |  |  |  |
| Thr | ACU* | 1.46(3527) | 0.73(1823) |
|  | ACC | 0.41(981) | 1.83(4546) |
|  | ACA* | 1.93(4683) | 0.50(1238) |
|  | ACG | 0.20(496) | 0.94(2350) |
|  |  |  |  |
| Ala | GCU* | 1.55(5779) | 0.84(2939) |
|  | GCC | 0.41(1546) | 1.84(6484) |
|  | GCA* | 1.90(7090) | 0.50(1756) |
|  | GCG | 0.14(532) | 0.82(2889) |
|  |  |  |  |
| Cys | UGU* | 1.34(2241) | 0.48(703) |
|  | UGC | 0.66(1112) | 1.52(2245) |
| Ter | UGA | 1.19(326) | 1.06(300) |
| Trp | UGG | 1.00(2367) | 1.00(1942) |
|  |  |  |  |
| Arg | CGU | 0.94(1690) | 0.87(1162) |
|  | CGC | 0.37(661) | 1.87(2499) |
|  | CGA* | 0.86(1547) | 0.61(822) |
|  | CGG | 0.52(930) | 0.79(1055) |
|  | AGA* | 1.95(3511) | 0.81(1079) |
|  | AGG* | 1.37(2469) | 1.05(1404) |
|  |  |  |  |
| Gly | GGU* | 1.33(4332) | 0.89(2639) |
|  | GGC | 0.52(1673) | 1.74(5172) |
|  | GGA* | 1.50(4877) | 0.70(2079) |
|  | GGG | 0.65(2110) | 0.68(2021) |

Notes:

- Codons showing significantly higher frequencies in highly- expressed genes (P<0.001).

Table S5. Over-representation of functional categories among *Picea* genes in large gene families

| GO term | GO-ID | Adjusted P-value (FDR<0.05) | No. of *Picea* genes in big families | Total no. of *Picea* genes |
| --- | --- | --- | --- | --- |
| protein kinase activity | 4672 | 0.0000E-100 | 224 | 914 |
| phosphotransferase activity, alcohol group as acceptor | 16773 | 0.0000E-100 | 224 | 999 |
| phosphorylation | 16310 | 0.0000E-100 | 200 | 859 |
| kinase activity | 16301 | 0.0000E-100 | 229 | 1286 |
| phosphate metabolic process | 6796 | 3.0000E-100 | 200 | 941 |
| phosphorus metabolic process | 6793 | 4.0000E-100 | 200 | 943 |
| transferase activity, transferring phosphorus-containing groups | 16772 | 2.0915E-86 | 230 | 1566 |
| protein serine/threonine kinase activity | 4674 | 3.1894E-68 | 122 | 449 |
| binding | 5488 | 2.1444E-49 | 344 | 5263 |
| nucleotide binding | 166 | 1.4177E-46 | 190 | 1776 |
| transferase activity | 16740 | 1.0412E-43 | 233 | 2718 |
| protein amino acid phosphorylation | 6468 | 6.7036E-39 | 95 | 504 |
| molecular_function | 3674 | 2.1242E-37 | 446 | 9821 |
| catalytic activity | 3824 | 1.2855E-36 | 373 | 6835 |
| stamen development | 48443 | 2.9325E-29 | 45 | 121 |
| androecium development | 48466 | 2.9325E-29 | 45 | 121 |
| macromolecule modification | 43412 | 7.7437E-28 | 140 | 1440 |
| flower development | 9908 | 1.6943E-23 | 68 | 421 |
| MAPKKK cascade | 165 | 3.0823E-22 | 37 | 112 |
| post-translational protein modification | 43687 | 1.2887E-21 | 105 | 1025 |
| signal transduction | 7165 | 1.9288E-21 | 73 | 529 |
| intracellular protein kinase cascade | 7243 | 3.4973E-21 | 37 | 121 |
| regulation of biological process | 50789 | 6.2092E-21 | 180 | 2544 |
| signal transmission | 23060 | 9.4146E-21 | 74 | 560 |
| signaling process | 23046 | 9.4146E-21 | 74 | 560 |
| mRNA modification | 16556 | 1.2811E-20 | 31 | 80 |
| signal transmission via phosphorylation event | 23014 | 1.8300E-20 | 37 | 128 |
| endonuclease activity | 4519 | 4.5645E-20 | 29 | 70 |
| signaling | 23052 | 7.7221E-20 | 107 | 1124 |
| response to salicylic acid stimulus | 9751 | 1.2890E-19 | 47 | 236 |
| receptor signaling protein activity | 5057 | 1.9114E-19 | 23 | 38 |
| receptor signaling protein serine/threonine kinase activity | 4702 | 1.9114E-19 | 23 | 38 |
| floral whorl development | 48438 | 4.2483E-19 | 51 | 290 |
| response to molecule of bacterial origin | 2237 | 4.3926E-19 | 24 | 45 |
| regulation of cellular process | 50794 | 6.6838E-19 | 153 | 2070 |
| cellular metabolic process | 44237 | 1.7995E-18 | 287 | 5409 |
| protein modification process | 6464 | 1.6326E-17 | 109 | 1251 |
| reproductive structure development | 48608 | 2.2247E-17 | 94 | 983 |
| intracellular signal transduction | 35556 | 4.9671E-17 | 48 | 289 |
| floral organ development | 48437 | 6.5248E-17 | 52 | 342 |
| response to bacterium | 9617 | 1.4942E-16 | 46 | 273 |
| intracellular signaling pathway | 23034 | 2.4604E-16 | 53 | 367 |
| peroxidase activity | 4601 | 2.1391E-15 | 26 | 82 |
| oxidoreductase activity, acting on peroxide as acceptor | 16684 | 2.1391E-15 | 26 | 82 |
| signaling pathway | 23033 | 2.5462E-15 | 82 | 847 |
| biological regulation | 65007 | 4.0994E-15 | 190 | 3138 |
| metabolic process | 8152 | 4.9188E-15 | 319 | 6626 |
| nuclease activity | 4518 | 9.0946E-15 | 29 | 114 |
| antioxidant activity | 16209 | 9.8020E-15 | 26 | 88 |
| post-embryonic organ development | 48569 | 9.8020E-15 | 55 | 432 |
| signal transducer activity | 4871 | 1.4003E-14 | 40 | 235 |
| molecular transducer activity | 60089 | 1.4003E-14 | 40 | 235 |
| reproductive developmental process | 3006 | 4.5917E-14 | 95 | 1135 |
| regulation of meristem development | 48509 | 7.2485E-14 | 32 | 155 |
| post-embryonic development | 9791 | 7.7691E-14 | 100 | 1242 |
| stomatal complex development | 10374 | 1.1283E-13 | 28 | 117 |
| regulation of meristem growth | 10075 | 1.1439E-13 | 30 | 137 |
| mRNA metabolic process | 16071 | 1.1594E-13 | 38 | 227 |
| reproductive process | 22414 | 5.9640E-13 | 96 | 1206 |
| protein tyrosine kinase activity | 4713 | 7.6099E-13 | 25 | 98 |
| stomatal complex morphogenesis | 10103 | 7.6099E-13 | 25 | 98 |
| shoot system development | 22621 | 1.0649E-12 | 54 | 472 |
| shoot development | 48367 | 1.0649E-12 | 54 | 472 |
| reproduction | 3 | 1.0936E-12 | 96 | 1220 |
| monooxygenase activity | 4497 | 1.9048E-12 | 29 | 143 |
| defense response to nematode | 2215 | 8.3225E-12 | 11 | 11 |
| MAP kinase activity | 4707 | 8.3225E-12 | 11 | 11 |
| response to jasmonic acid stimulus | 9753 | 1.4802E-11 | 38 | 268 |
| meristem maintenance | 10073 | 2.1538E-11 | 32 | 194 |
| RNA modification | 9451 | 2.1541E-11 | 31 | 182 |
| anther development | 48653 | 4.1674E-11 | 20 | 71 |
| response to other organism | 51707 | 4.7074E-11 | 76 | 912 |
| oxidoreductase activity, acting on paired donors, with incorporation or reduction of molecular oxygen | 16705 | 5.9680E-11 | 33 | 215 |
| iron ion binding | 5506 | 5.9680E-11 | 34 | 228 |
| regulation of innate immune response | 45088 | 8.3691E-11 | 33 | 218 |
| regulation of immune system process | 2682 | 1.0338E-10 | 33 | 220 |
| regulation of immune response | 50776 | 1.0338E-10 | 33 | 220 |
| regulation of developmental growth | 48638 | 1.7904E-10 | 31 | 199 |
| detection of biotic stimulus | 9595 | 2.3150E-10 | 17 | 53 |
| regulation of growth | 40008 | 4.5890E-10 | 31 | 207 |
| meristem development | 48507 | 4.9193E-10 | 39 | 319 |
| post-embryonic morphogenesis | 9886 | 9.4861E-10 | 37 | 297 |
| regulation of programmed cell death | 43067 | 9.5292E-10 | 32 | 227 |

Table S6. Over-representation of functional categories among *Picea* genes in single-copy gene families

| GO term | GO-ID | Adjusted P-value (FDR<0.05) | No. of *Picea* single-copy genes | Total no. of *Picea* genes |
| --- | --- | --- | --- | --- |
| nucleobase, nucleoside, nucleotide and nucleic acid metabolic process | 6139 | 3.6236E-59 | 264 | 1649 |
| cellular nitrogen compound metabolic process | 34641 | 1.1583E-57 | 300 | 2128 |
| intracellular | 5622 | 2.3325E-57 | 570 | 6668 |
| nitrogen compound metabolic process | 6807 | 1.7355E-56 | 302 | 2187 |
| nucleic acid metabolic process | 90304 | 1.4867E-53 | 213 | 1192 |
| cellular macromolecule metabolic process | 44260 | 1.3065E-51 | 355 | 3052 |
| cellular metabolic process | 44237 | 6.7892E-50 | 493 | 5409 |
| gene expression | 10467 | 1.7948E-49 | 181 | 931 |
| macromolecule metabolic process | 43170 | 1.9708E-48 | 368 | 3346 |
| primary metabolic process | 44238 | 1.2592E-47 | 469 | 5056 |
| RNA metabolic process | 16070 | 7.6513E-46 | 166 | 840 |
| intracellular part | 44424 | 5.0308E-44 | 527 | 6302 |
| cellular process | 9987 | 4.6462E-43 | 568 | 7200 |
| metabolic process | 8152 | 2.4288E-37 | 529 | 6626 |
| cell part | 44464 | 3.5206E-35 | 592 | 8084 |
| cell | 5623 | 3.8389E-35 | 592 | 8087 |
| cellular_component | 5575 | 9.3187E-35 | 594 | 8152 |
| intracellular organelle | 43229 | 2.5536E-34 | 467 | 5561 |
| cellular biosynthetic process | 44249 | 2.5536E-34 | 296 | 2731 |
| organelle | 43226 | 2.5536E-34 | 467 | 5563 |
| biosynthetic process | 9058 | 8.4114E-32 | 302 | 2908 |
| cytoplasm | 5737 | 5.9065E-31 | 450 | 5403 |
| intracellular membrane-bounded organelle | 43231 | 1.2751E-29 | 442 | 5320 |
| membrane-bounded organelle | 43227 | 1.2835E-29 | 442 | 5321 |
| RNA processing | 6396 | 2.4718E-29 | 104 | 500 |
| cellular macromolecule biosynthetic process | 34645 | 3.2850E-29 | 165 | 1146 |
| intracellular organelle part | 44446 | 1.1576E-28 | 225 | 1920 |
| organelle part | 44422 | 1.2024E-28 | 225 | 1921 |
| macromolecule biosynthetic process | 9059 | 5.5297E-28 | 168 | 1212 |
| cytoplasmic part | 44444 | 2.7370E-27 | 424 | 5112 |
| small molecule metabolic process | 44281 | 4.5013E-26 | 254 | 2415 |
| macromolecular complex | 32991 | 7.5655E-24 | 148 | 1077 |
| protein metabolic process | 19538 | 1.7611E-23 | 226 | 2110 |
| cellular protein metabolic process | 44267 | 6.5479E-23 | 211 | 1921 |
| translation | 6412 | 2.7155E-22 | 74 | 331 |
| organelle organization | 6996 | 6.3458E-22 | 157 | 1240 |
| biological_process | 8150 | 1.3040E-21 | 630 | 9688 |
| cellular component organization | 16043 | 2.3299E-21 | 202 | 1853 |
| cellular nitrogen compound biosynthetic process | 44271 | 1.0655E-20 | 100 | 609 |
| chloroplast | 9507 | 1.2817E-19 | 169 | 1467 |
| plastid | 9536 | 3.0659E-19 | 203 | 1947 |
| ncRNA metabolic process | 34660 | 3.1050E-16 | 50 | 206 |
| binding | 5488 | 1.2901E-15 | 396 | 5263 |
| chloroplast part | 44434 | 1.6693E-15 | 102 | 751 |
| structural constituent of ribosome | 3735 | 2.8763E-15 | 49 | 210 |
| mitochondrion | 5739 | 2.8763E-15 | 122 | 1002 |
| structural molecule activity | 5198 | 3.8748E-15 | 56 | 273 |
| plastid part | 44435 | 5.5740E-15 | 102 | 766 |
| molecular_function | 3674 | 1.5809E-14 | 618 | 9821 |
| ribonucleoprotein complex biogenesis | 22613 | 5.6085E-14 | 44 | 185 |
| protein complex | 43234 | 5.6085E-14 | 91 | 664 |
| RNA binding | 3723 | 5.7790E-14 | 49 | 228 |
| ribosome biogenesis | 42254 | 1.4162E-13 | 43 | 182 |
| chloroplast stroma | 9570 | 2.2146E-13 | 66 | 402 |
| cellular component biogenesis | 44085 | 2.6360E-13 | 100 | 791 |
| plastid stroma | 9532 | 3.7708E-13 | 66 | 407 |
| ribonucleoprotein complex | 30529 | 5.7406E-13 | 59 | 339 |
| non-membrane-bounded organelle | 43228 | 6.2848E-13 | 93 | 718 |
| intracellular non-membrane-bounded organelle | 43232 | 6.2848E-13 | 93 | 718 |
| methylation | 32259 | 1.8942E-12 | 57 | 329 |
| ribosome | 5840 | 2.0453E-12 | 51 | 271 |
| RNA modification | 9451 | 2.5154E-12 | 41 | 182 |
| alcohol metabolic process | 6066 | 3.2399E-12 | 82 | 608 |
| monosaccharide metabolic process | 5996 | 4.4831E-12 | 70 | 475 |
| establishment of localization in cell | 51649 | 4.4831E-12 | 103 | 869 |
| RNA methylation | 1510 | 1.0304E-11 | 26 | 75 |
| cellular localization | 51641 | 1.5147E-11 | 104 | 900 |
| nucleus | 5634 | 1.6514E-11 | 143 | 1428 |
| cellular ketone metabolic process | 42180 | 2.8107E-11 | 131 | 1271 |
| nucleobase, nucleoside, nucleotide and nucleic acid biosynthetic process | 34654 | 3.6105E-11 | 40 | 190 |
| nucleobase, nucleoside and nucleotide biosynthetic process | 34404 | 3.6105E-11 | 40 | 190 |
| ncRNA processing | 34470 | 3.8853E-11 | 36 | 156 |
| cellular carbohydrate metabolic process | 44262 | 4.4527E-11 | 113 | 1036 |
| RNA splicing | 8380 | 4.5255E-11 | 36 | 157 |
| oxoacid metabolic process | 43436 | 4.8222E-11 | 129 | 1255 |
| carboxylic acid metabolic process | 19752 | 4.8222E-11 | 129 | 1255 |
| organic acid metabolic process | 6082 | 5.0270E-11 | 129 | 1256 |
| nucleotide biosynthetic process | 9165 | 5.2035E-11 | 39 | 184 |
| macromolecule methylation | 43414 | 7.4998E-11 | 48 | 271 |
| one-carbon metabolic process | 6730 | 9.8006E-11 | 57 | 366 |
| cytosol | 5829 | 1.2731E-10 | 124 | 1205 |
| mRNA metabolic process | 16071 | 1.2890E-10 | 43 | 227 |
| catabolic process | 9056 | 1.4443E-10 | 131 | 1305 |
| nucleobase, nucleoside and nucleotide metabolic process | 55086 | 1.4443E-10 | 72 | 539 |
| intracellular transport | 46907 | 1.5478E-10 | 91 | 772 |
| macromolecule modification | 43412 | 2.0867E-10 | 140 | 1440 |
| envelope | 31975 | 2.7111E-10 | 72 | 547 |
| heterocycle metabolic process | 46483 | 3.3221E-10 | 85 | 708 |
| nucleic acid binding | 3676 | 3.6743E-10 | 135 | 1381 |
| DNA metabolic process | 6259 | 3.6978E-10 | 59 | 402 |
| small molecule biosynthetic process | 44283 | 6.1962E-10 | 123 | 1223 |
| organelle envelope | 31967 | 6.1962E-10 | 71 | 546 |
| cellular macromolecule catabolic process | 44265 | 6.7559E-10 | 53 | 343 |
| cellular aldehyde metabolic process | 6081 | 9.3151E-10 | 38 | 195 |
| macromolecule catabolic process | 9057 | 9.7238E-10 | 60 | 424 |

Figure S1. Plot of axis 1 to 4 of a correspondence analysis of codon usage in 26,052 genes from *P. abies*. Black circles represent codons from highly expressed genes, and red circles, codons from lowly expressed genes.

Figure S2. Plot of axis 1 to 4 of a correspondence analysis of codon usage in 19,056 genes from *P. glauca*. Black circles represent codons from highly expressed genes, and red circles, codons from lowly expressed genes.

Figure S3. Frequency distribution of gene family size in *P.glauca* and *P.abies*. Each dot represents one orthologous gene family.

Figure S4. Gene family size showed significant associations with expression level, expression breadth, GC content at third position and codon bias (Fop) in *P. glauca*.

Figure S5. Number of synonymous nucleotide substitutions (dS) and non-synonymous nucleotide substitutions (dN) vs. gene family size

Figure S6. Heatmap showing gene expression profiles of orthologous single copy genes in *P. glauca*. Each row is a single copy gene, and each column is a tissue. Expression levels go from 0 (lowly expressed) to 10 (highly expressed). Tissues studied included buds (b), needles (n), wood-early (we), wood-late (wl), megagametophytes (meg), phelloderm (phe), adventitious roots (ro) and embryonic cells (ec).

Figure S7. Plant Terpenoid simplified pathway, modified from Ramsay et al. 2009. Numbers indicate pathway positions in each of the studied branches.

Figure S8. Sequence logo showing motif enrichment in the three most statistically significant motifs (from top to bottom) in the Promoter region of genes pertaining to two *Picea abies* gene families: A) Gene family “Picea 1012”, containing 118 genes, and annotated as MYb-like DNA-binding domain (PF00249.26, PF1392.1); and B) Gene family “Picea 1028”, containing 62 genes, annotated as Pectinesterase (PF01095.14; PF04043.10). The height of the motif block is proportional to –log (p-value), truncated as the height for a motif with a p-value of 1e-10. Sequence logos were obtained using MEME (Bailey et al. 2009).

Figure S9. Figure shows A) sequence logo showing motif enrichment in the three most statistically significant motifs (from top to bottom) in the Promoter region of genes pertaining to *Picea abies* gene family “Picea 1236”, containing 23 genes, annotated as Fasciclin (PF02469.17). Sequence logos were obtained using MEME (Bailey et al. 2009). B) Maximum-Likelihood phylogenetic tree with 100 bootstrap replicates showing all orthologous and paralogous genes for *P. abies* and *P. glauca*, for the same gene family “Picea 1236”. Phylogenetic tree was constructed using MEGA 6.06 (Tamura et al. 2013).
